# Hemorheological and Microcirculatory Factors in Liver Ischemia-Reperfusion Injury—An Update on Pathophysiology, Molecular Mechanisms and Protective Strategies

**DOI:** 10.3390/ijms22041864

**Published:** 2021-02-13

**Authors:** Norbert Nemeth, Katalin Peto, Zsuzsanna Magyar, Zoltan Klarik, Gabor Varga, Mihai Oltean, Anna Mantas, Zoltan Czigany, Rene H. Tolba

**Affiliations:** 1 Department of Operative Techniques and Surgical Research, Faculty of Medicine, University of Debrecen, H-4032 Debrecen, Hungary; kpeto@med.unideb.hu (K.P.); zsuzsa.magyar@med.unideb.hu (Z.M.); zklarik@gmail.com (Z.K.); gaborvarga@med.unideb.hu (G.V.); 2 The Transplant Institute, Sahlgrenska University Hospital, 413 45 Gothenburg, Sweden; mihai.oltean@surgery.gu.se; 3 Department of Surgery and Transplantation, Faculty of Medicine, RWTH-Aachen University, 52047 Aachen, Germany; amantas@ukaachen.de (A.M.); zczigany@ukaachen.de (Z.C.); 4 Institute for Laboratory Animal Science and Experimental Surgery, Faculty of Medicine, University Hospital RWTH-Aachen, 52047 Aachen, Germany; rtolba@ukaachen.de

**Keywords:** hepatic ischemia-reperfusion, hemorheology, microcirculation, preventive and therapeutic strategies

## Abstract

Hepatic ischemia-reperfusion injury (IRI) is a multifactorial phenomenon which has been associated with adverse clinical outcomes. IRI related tissue damage is characterized by various chronological events depending on the experimental model or clinical setting. Despite the fact that IRI research has been in the spotlight of scientific interest for over three decades with a significant and continuous increase in publication activity over the years and the large number of pharmacological and surgical therapeutic attempts introduced, not many of these strategies have made their way into everyday clinical practice. Furthermore, the pathomechanism of hepatic IRI has not been fully elucidated yet. In the complex process of the IRI, flow properties of blood are not neglectable. Hemorheological factors play an important role in determining tissue perfusion and orchestrating mechanical shear stress-dependent endothelial functions. Antioxidant and anti-inflammatory agents, ischemic conditioning protocols, dynamic organ preservation techniques may improve rheological properties of the post-reperfusion hepatic blood flow and target endothelial cells, exerting a potent protection against hepatic IRI. In this review paper we give a comprehensive overview of microcirculatory, rheological and molecular–pathophysiological aspects of hepatic circulation in the context of IRI and hepatoprotective approaches.

## 1. Introduction

Liver ischemia-reperfusion injury (IRI) remains inevitable during extended liver resections and orthotopic liver transplantation (OLT). Hepatic IRI is a multifactorial phenomenon which has been associated with adverse clinical outcomes [1,2,3,4].

Ischemia-reperfusion injury-related tissue damage is characterized by various chronological events depending on the experimental model or clinical setting [4,5,6]. In major hepatectomies, a shorter phase of warm ischemia (e.g., intermittent or continuous Pringle/Baron maneuver) is followed by normothermic in situ reperfusion. In the setting of OLT, the extent of IR-related damage is significantly dependent on the type of organ donation. In a donation after brain death (DBD) cold ischemia is followed by a warm reperfusion in the recipient. The quality of the allograft, the length of cold storage and the time required for the implantation of the allograft are major determinants of clinical outcomes, especially in marginal DBD allografts [4]. In donation after circulatory death (DCD) donors, an additional period of in situ warm ischemia in the donor exposes the recipients to a higher risk of graft-related complications such as an increased risk of biliary complications (biliary stenosis, ischemic-type biliary lesions) [4].

**Figure 1:**
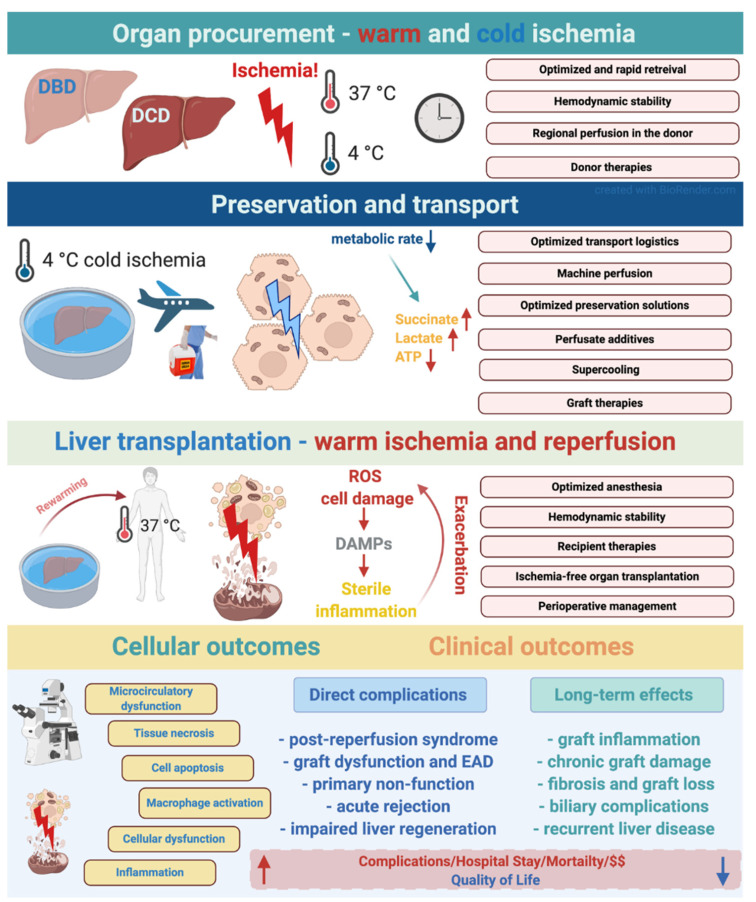
Various phases of ischemia reperfusion injury and their effects and mechanisms in the setting of deceased donor liver transplantation. Potential stages and interventional strategies to mitigate IRI during organ transplantation logistics. The figure was created with BioRender (https://biorender.com).

The extent of IRI has been associated with a broad spectrum of complications and inferior outcomes in OLT and partial hepatectomies. IRI has been demonstrated to be a major risk-factor for perioperative morbidity, early allograft dysfunction (EAD) and primary non-function in liver transplantation [4]. Besides its short-term effects, IRI also has long-term consequences leading to graft injury, fibrosis, rejection and biliary injury [3,7] (Figure 1). 

**Figure 2:**
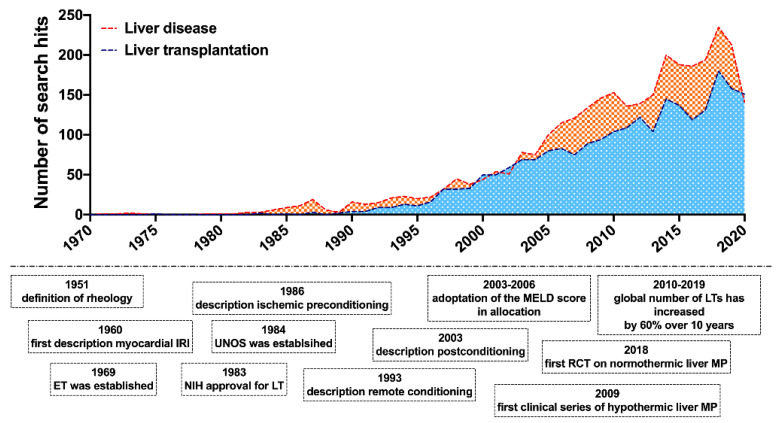
Major milestones in the history of hemorheology, ischemia-reperfusion and liver transplantation research. Publication activity in the field of rheological and ischemia-reperfusion injury research in liver disease and liver transplantation. Number of corresponding search hits in liver disease and liver transplantation in the PubMed^®^ database. Accessed on 13th December 2020; search syntax: ((ischemia-reperfusion) OR (rheology)) AND (liver transplantation) // ((ischemia-reperfusion) OR (rheology)) AND (liver disease).

IRI research has continuously drawn scientific interest with a significant and continuous increase in publication activity over the years (Figure 2) and a large number of pharmacological and surgical therapeutic attempts suggested [4,5,8,9,10]. Nonetheless, few therapeutic interventions have made their way into everyday clinical practice.

In recent decades extensive research has been conducted on cellular lesions and various signaling processes, from inflammatory mechanisms, and immune responses to metabolic and systemic changes, including alterations in remote organs’ function [1,11,12,13,14,15,16,17,18,19,20,21]. There is also growing knowledge about hemodynamic changes and the dynamics of microcirculation in general, however, there is another factor about which is discussed relatively little, and that is the rheology of the circulating blood itself [22,23,24,25,26,27]. Through its macro- and micro-rheological properties, blood plays an important role in modulating not only the determinant factors of tissue perfusion but also the shear stress mediated processes in the endothelial cells [28,29,30,31]. Accordingly, these are the elements of the complex etiologic mechanism of ischemia-reperfusion events that cannot be ignored.

In the present review we present a comprehensive overview on the significance and mechanisms of hepatic IRI with focus on microcirculatory and rheological aspects. Furthermore, various therapeutic interventions are discussed.

## 2. Rheology of the Blood—A Brief Overview

Circulation, tissue perfusion, endothelial/vascular function: all are strongly linked to the rheological features of the blood [32]. The main parameters determining blood viscosity are the plasma viscosity, the hematocrit, and the micro-rheological factors, such as red blood cell deformability and aggregation [23,24,25]. All of these affect flow conditions and perfusion, showing a great complexity in the circulatory bed. Pressure (pressure gradient), shear rate—shear stress, velocity of flow, vascular resistance and impedance count in biomechanics of the blood stream. Several factors influence the blood flow in vivo, and estimating the flow conditions in large vessels using the major hydrodynamic and physical fluid flow equations can be only partially used. Therefore, a single constitutive equation is not appropriate to fully understand blood rheology. However, several approaches exist to define these conditions (e.g., Newtonian and non-Newtonian fluid, the Einstein model, Bingham fluid model, Casson model, Quemada model) [24,25,33]. Below, we summarize the major aspects.

### 2.1. Biomechanical, Cellular and Molecular Aspects

In non-Newtonian fluids, such as the blood, the viscosity increases with decreasing shear rate (the relation of shear stress and shear rate is non-linear: Casson-curve). Furthermore, in blood there is a minimal shear stress that is required to start the flow. It is called yield-stress. It is well known that the shear rate and shear stress significantly vary along the circulation with the highest shear stress occurring in the arterioles and capillaries [33,34,35].

In case of fully laminar flow, due to the friction, low velocity occurs at the tube (vessel) wall, and the highest flow velocity can be observed along the axis, resulting in a parabolic velocity profile in the longitudinal cross-section of the tube. The tangent of the streamlines reflects the flow direction at a given point. The concentration of the streamlines determines the magnitude of the flow. At the vicinity of the tube wall the flow is dominantly laminar, while along the axis the velocity is higher and its homogeneity is smaller, with turbulences developing as a consequence. This is due to the unbalance between inertial and frictional forces when velocity increases. Turbulence starts above ~2100 Re (real turbulence: Re > 10^4^). Real turbulence—by definition—does not occur in the vasculature. The critical velocity shows when the flow becomes turbulent: ν_crit_ = Re ηρ [24,25,33].

The Δp versus Q curve is almost linear below 1000 Re (Q ~ Δp); above it the curve starts to flatten (Q ~ √Δp): when increasing the pressure-gradient the flow-increase does not increase in the same manner and, due to the developing turbulence, the flow resistance increases [36].

Further effects occurring include Dean-vortices and the Magnus-effect. In the circulation the pulsatility also takes part in forming the flow profile, showing a decreasing effect toward the capillaries. The Wommersley-number reflects the pulsatility: α = R (ωρ/η)^1/2^, where R: radius, ω: heart frequency ρ: blood density, η: blood viscosity. Pressure-oscillation also has to be mentioned here, which is composed of various frequency components: first-order (pulsating), second-order (respiration-related), third-order (Traube–Hering–Mayer) waves. The characteristics of blood flow are determined not only by the properties of the circulating blood, but also by the geometry of the vascular system, its regulatory mechanisms, and the passive biomechanics of the vessel wall.

Numerous endothelial functions are modulated by the shearing force, so by the rheological properties of the blood and the flow profile: membrane ion channel proteins, receptor-dependent G-proteins, protein kinase cascades, transcription factors, such as NF-κB, BGR-1, c-FOS, among others. Cytoskeleton, membrane caveolae, junctional proteins, adhesion complexes also take part in shear-induced endothelial responses. Fluid shear stress plays an important role in physiological and pathological vascular remodeling as well, via junctional mechanosensory complex (PECAM-1, VEGFRs, VE-Cad) and CCM complexes (CCM1, CCM2, CCM3). Consequently, any changes in blood rheology (increased blood and/or plasma viscosity, impaired red blood cell deformability, enhanced red blood cell aggregation) strongly affect endothelial functions [28,29,30,31,37].

### 2.2. Blood and Plasma Viscosity

The blood is a non-Newtonian, thixotropic and viscoelastic fluid. Its viscosity depends on the shear rate. The non-Newtonian characteristic of the blood is hematocrit and shear rate dependent. At lower shear rate the blood viscosity increases due to the viscosity elevating effect of the red blood cell aggregation. At higher flow velocity (i.e., higher shear rate), the cells disaggregate and elongate in the direction of the flow due to their deformability and elastic features [24,25,33]. The connection between blood viscosity and hematocrit (Htc) shows an exponential-like rather than a linear relation [24]. The hematocrit/viscosity rate presented in the function of the hematocrit shows a connection resembling of a bell-shaped curve, with a peak (by derivation of the curve), which reflects the maximum of oxygen transporting capacity of blood. It is concerned as an “optimal” hematocrit, i.e., the possible highest red blood cell count (the highest hematocrit) at the possible lowest viscosity [38]. 

Plasma viscosity is primarily determined by its water content and the macromolecules with elongated configuration, such as certain plasma proteins (fibrinogen, globulin fractions), besides triglycerides and lipoproteins [25]. Along the vasculature the apparent viscosity of the blood is not constant due to the intravascular interactions and distribution of different shear rate profile and the formed elements, mainly the red blood cells; however, the intravascular plasma viscosity can be considered constant. Consequently, the plasma viscosity maintains shear stress on the endothelium at the cell-poor or often cell-free zone in the direct vicinity of the endothelium (Poiseuille-zone), due to the axial flow profile in vessels with diameter approximately under 300 μm [25].

### 2.3. Red Blood Cell Deformability

The ability of passive deformation of the red blood cells by shearing and compressing forces depends on the absolute volume, surface/volume ratio of the cells, morphological characteristics, viscoelastic properties of the cell membrane and intracellular viscosity [24,25,39,40,41,42].

These features are dominantly derived from the molecular composition of the cell membrane, cytoskeletal structure. More than 50 transmembrane proteins are known in erythrocytes: transport proteins (anion transporter band-3, water transporter aquaporin-1, glucose and l-dehydroascorbinic acid transporter glut1, urea transporter Kidd antigen protein, transporter RhAG, Na^+^/K^+^-ATPase, Ca^2+^ATPase, Na^+^-K^+^-2Cl^−^, Na^+^-Cl^−^, Na^+^-K^+^, K^+^-Cl^−^ co-transporters, Gárdos-channel), adhesion molecules, receptor, blood group antigens, A–D glycophorin complexes, and other proteins. This structure gives the red blood cells’ membrane elasticity and mechanical stability. From pathophysiological point of view, it is important to mention that this stability depends on the composition of the proteins, phosphorilation state, intracellular Ca^2+^ concentration, and free-radical reactions affect their structure and function. More than 340 membrane proteins are known. Their complexity is high, about 80% of them are still not associated with structural–functional models; their roles have not been fully elucidated yet [43,44].

Mammalian mature red blood cells are unnucleated, so the intracellular viscosity is mainly determined by the properties of hemoglobin content. Mean cell hemoglobin concentration is regulated within a relatively narrow range (30−35 g/d). It is also well-known that ATP-dependent Na^+^, K^+^ transporters participate in the cell volume regulation [23,25,39,44,45].

Viscosity property derives from membrane viscosity and cytoplasm viscosity, and elasticity characteristic is associated with the surface expansion, shearing and bending elements [46]. Motion of red blood cells depends on the flow conditions: rolling, rotation, swinging, elongation, elastic deformation, the combination of all the above [34,35,47]. Worsening deformability, i.e., the enhanced rigidity of red blood cells, in the mass flow zone of circulation (vessel diameter >300 μm) may lead to elevated blood viscosity. Red blood cells with reduced deformability can cause the most significant problem in the microcirculation, more precisely in the zone of so-called individual flow. Here, capillaries with diameter of 3–5 μm may also occur. Deformability is essential to passing through these capillaries.

### 2.4. Red Blood Cell Aggregation

Under stasis or at low shear rate erythrocytes reversibly clump together. Initially they arrange next to each other in a rouleaux formation, resembling a stack of coins. It happens within a few seconds (1–5 s). The rouleaux form larger two- then three-dimensional shape aggregates in the next seconds or even minutes [23]. In anticoagulated blood samples due to the gravity these aggregates show sedimentation. If the aggregation is fast and/or extensive, thus the erythrocyte sedimentation rate (ESR) values are higher.

Red blood cell aggregability is influenced by cellular factors. The aggregation happens when plasmatic factors are also presented. The process of red blood cell aggregation is not completely understood yet. There are two theories. (1) Bridging model: the aggregation happens via non-covalent cross-linking of macromolecules (large proteins with elongated structure, such as fibrinogen, or in vitro with different polymers). (2) Depletion model: since the glycocalyx layer does not allow the macromolecules to penetrate close to the membrane, a depletion zone develops in the vicinity of the cells. As two red blood cells approach each other, due to the osmotic gradient between the macromolecule concentration of the plasma phase and the depletion zone, a kind of “pulling” force may develop [23]. Disaggregating forces include electrostatic repulsion (due to surface negative charge), membrane strain, and shearing forces. There is no clear consensus about these two theories. Maybe both might be true. Carvalho et al. using atomic-force microscopy showed that the α_IIb_β_3_ glycoprotein complexes of erythrocytes (similar exist on platelets’ surface) have lower affinity to link fibrinogen. It is supposed that the depletion forces are important to “pull” the cells close enough to each other, and so weak linking may happen [48].

Various plasma proteins and in vitro different polymers of different molecular size influence the aggregation [23]. Fibrinogen, C-reactive protein and immunoglobulin M facilitate and enhance red blood cell aggregation. Immunoglobulin G and haptoglobin show rather increasing effects, while transferrin, coeruloplasmin and albumin do not influence erythrocyte aggregation [23]. In vitro high molecular weight macromolecules and polymers (e.g., 60 or 73 kDa dextran, 360 kDa polyvinylpyrrolidon, 36 kDa polyethylen-glycol) promote red blood cell aggregation. Armstrong et al. found that enhanced red blood cell aggregation was observed if the hydrodynamic radius (R_h_) of the macromolecules/polymers was larger than 4 nm. Molecules with smaller R_h_ did not influence the aggregation process [49].

Red blood cell aggregation depends on the hematocrit (Hct) as well. However, the relationship is not linear. The aggregation index, which increases by the extent of aggregation, expresses a nearly linear relation between ~20 and 40% Hct, between about 40 and 50% the slope of the curve is smaller, while at higher Hct the aggregation does not change significantly with further increase in the Hct [23]. For aggregation the biconcave cell morphology is crucial, since ovalocyte, spherocyte, echinocyte, sphero-echinocyte cell forms are less capable of aggregating [23,26]. Due to the complex background given by the cellular and plasmatic factors, red blood cell aggregation shows the highest diversity amongst animal species compared to human [23,26,50,51].

### 2.5. Hemorheological Factors in Microcirculation

**Figure 3:**
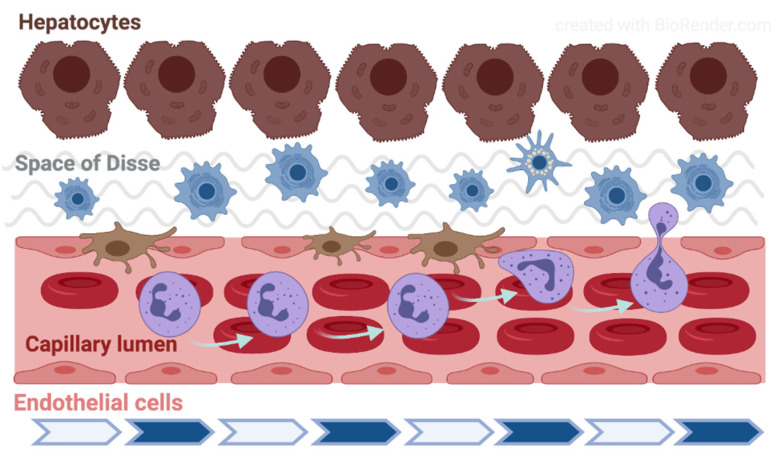
Schematic drawing of the liver microcirculation. The figure was created with BioRender (https://biorender.com).

Fåhræus observed in 1958 that by enhanced aggregation the axial flow in the glass capillary is more expressed and the cell-free side zone is wider [52]. The parabolic flow rate profile and the presence of circulating blood cells lead to the phenomenon that typically appears in the range under 200–300 µm vein diameter: the axial migration of red blood cells. Along the vessel’s cross section, the distribution and velocity of red blood cells lead to the dynamic reduction in hematocrit (Fåhræus-effect). Below approximately 30 µm diameter, down to about 10 µm, the apparent blood viscosity decreases with the diameter (Fåhræus–Lindqvist effect) [53]. Along the vessel wall the cell-poor/cell-free Poiseuille-zone reduces the friction, and thereby the hydrodynamic resistance. When the shear-rate enables the development of red blood cell aggregation (typically in the area of postcapillary venules), the aggregates also flow along the axis. Increased aggregation enlarges the circulating particles, so enhancing the axial migration. Consequently, the Poiseuille-zone widens which process facilitates the margination of leukocytes [54,55] (Figure 3).

In the microcirculation due to the extremely variable ramifications, connections, bends, endothelial surface features, red blood cell distribution, the tissue hematocrit is highly variable [34,35,56,57,58].

## 3. Characteristics of the Liver Circulation

### 3.1. Circulation and Microcirculation

The liver receives its blood supply both through the portal vein (around 75%) and the hepatic artery. These two vessels enter the liver and penetrate the liver parenchyma together and in close vicinity, and gradually branch until arterioles and venules. Upon reaching the periphery of the hexagonal liver lobules separately, the terminal portal venules enter the lobule in-between the cords of hepatocytes and continue with the sinusoids, a microvascular network which forms most of the capillary bed in the hepatic parenchyma [59,60,61]. The hepatic artery supplies oxygenated blood to the biliary tree (through the peribiliary plexus), portal tract interstitium and the vasa vasorum of the intrahepatic vessels. The arterioles also ultimately empty in the sinusoids resulting in mixing of arterial and portal blood. The sinusoids run straight for about 250 µm and converge radially towards the center of the lobule where all empty into the central vein. The sinusoids communicate with each other through shorter interconnecting sinusoids running across the liver cell and their diameter gradually increase from 5 to 7 µm in the periphery (portal zone) to 10 to 15 µm in the pericentral area [62] (Figure 3).

The ultrastructure of sinusoidal endothelial cells (SEC) differs greatly from other capillaries in the body due to the presence of sieve-like pores (or fenestrae) measuring between 100 and 200 nm and by the lack of a basement membrane [63]. These unique features result in a high, bidirectional permeability of the sinusoids to macromolecules, solutes and water. Besides the SEC, the microvascular network contains four other distinct components: the hepatic stellate cells (HSC), which are located in the space of Disse and modulate sinusoidal tone and stiffness; the Kupffer cells (KC), unique liver-resident macrophages anchored to the luminal site of the endothelium and exposed to the bloodstream; the hepatocytes, the parenchymal cells that are tightly attached to SECs and account for the liver metabolism; and the extracellular matrix (ECM), the scaffold of the entire structure, which serves as a niche for these cells and may influence their phenotype depending on its composition. SECs and KCs belong to the mononuclear phagocyte system (MPS), also known as the reticuloendothelial system. The majority of KCs are found in the peripheral, periportal area where they are larger and have greater phagocytic activity than those located in the perilobular region [62]. Both SECs and KCs are very sensitive to ischemia-reperfusion injury and are key elements in the local response after liver ischemia. Thus, the sinusoidal cells become swollen and may detach already during ischemia whereas KCs are activated during the early phase after reperfusion releasing free radicals, vasoactive mediators and proinflammatory cytokines in the ischemic areas [64,65]. The hepatic stellate cells reside outside the sinusoid, between the basal surfaces of the hepatocytes and the SEC, in the space of Disse [66]. In contrast to the KCs, HSCs are distributed homogeneously throughout the three zones of the liver lobule. Their finger-like projections called perisinusoidal processes surround the sinusoidal tube and may regulate its tone. HSCs produce various inflammatory molecules, interact with other liver cells and relay and integrate the signals from the sinusoids to the liver parenchyma. Hence, several cells may alter the delicate balance that maintains the microcirculatory homeostasis in the liver.

### 3.2. Rheological Differences (Aorto–Porto–Caval)

There is only limited information about the characteristics of micro-rheological parameters in different vascular segments [26,67]. It has been demonstrated that red blood cell aggregation and deformability show significant aorto–porto–caval differences in rats [68]. The smallest elongation index values were found in the arterial samples, the highest values were in the systemic venous blood and the values of the portal blood sample fall between the two. Son et al. also found in rats that elongation index values are lower in arterial blood compared to venous blood. These differences could not be observed in human or in canine blood [67]. The aggregation index parameters are significantly lower in the systemic venous blood, than in the arterial and portal samples [68]. The explanation of these arterio–venous or porta–caval micro-rheological heterogeneity might be derived from the dynamic differences in pH, oxygenation level, lactate concentration, hematocrit and mean cell volume [22,23,26,40,69].

## 4. Pathophysiology of Hemorheological Alterations Related to Hepatic Ischemia-Reperfusion

During IRI, metabolic alterations (decrease in pH, increase in H+ and lactate) and osmolarity changes affect the morphological and mechanical properties of blood cells [40,69,70,71]. A decrease in pH will transform the normally discocyte-shape red bloods cells into stomacyte or sphero-stomacyte morphology. If the ATP depletion and calcium accumulation are the dominant effects, the echinocyte and sphero-echinocyte morphologies appear. Red blood cells’ deformability is reduced, and their aggregation is disturbed in both morphological transformations [26,40,69,70]. Change in oxygenation is also known to alter micro-rheology, since deformability of deoxygenated red blood cells is impaired, being associated with enhanced aggregation. Under hypoxia, the cell swelling alters surface to volume ratio, and consequently cellular deformability decreases.

Conditions and circumstances that lead to IRI (e.g., clamping vessels, obturation/occlusion, intravascular devices) cause mechanical trauma to blood [22,26,72]. Blood cells are continuously exposed to mechanical stress during their entire life-span in the circulation. A shear stress ranging around 5–20 Pa may even improve their deformability by releasing NO from the cells, besides other vasoactive mediators [26,51,73,74,75,76,77]. Higher shear stress, correlating with the magnitude and exposure time, causes mechanical trauma to the blood cells from sublethal trauma (decreased deformability, enhanced aggregation) and microvesicle generation to mechanical hemolysis [51,72,78].

Free radicals can be generated during reperfusion of a previously damaged tissue by ischemia (xanthine oxidase/reductase, neutrophils, NO synthetase activity when forming OONO^−^ in the presence of NO and superoxide anion), and during the associated inflammatory reaction (neutrophils) [9,79]. Oxygen-centered free radicals can jeopardize red blood cells in several ways: lipid peroxidation on the cell membrane, sulfhydryl cross-linking and so altering structure and function of proteins (receptors, ion pumps, structural proteins), forming methemoglobin and Heinz-bodies. Red blood cells are very vulnerable for oxidative stress, because they are rich in iron (Fenton-reaction) and they do not have a nucleus; thus, they have no chance for new protein generation for any repair [9,22,27].

Due to inflammatory processes the developing acute phase reactions also cause non-specific hemorheological changes: elevated fibrinogen and α2-macroglobulin concentration accompanied by consequent increase in plasma viscosity, rise in leukocyte count, increase or decrease in platelet count, hemoconcentration, besides micro-rheological alterations [9,22,27]. Enhanced red blood cell aggregation elevates blood viscosity and increases the flow resistance: the axial migration of the cells becomes more expressed, widening Poiseuille-zone, facilitated leukocyte tethering and margination, slowed down rolling are also included [55].

Hypoxia leads to impaired endothelial cell barrier function and, additionally, altered blood rheology has an impact on the shear stress profile on the endothelial surface modulating numerous functions as discussed before. If hypovolemia is also associated with ischemia-reperfusion, due to the increased sympathetic activation vasoconstriction appears, leading to reduction in the capillary cross-sectional area and endothelial swelling. In the microcirculatory bed, the “no-reflow” phenomenon is characteristic for ischemia-reperfusion that is caused by microvascular spasm, swelling of endothelial cells, bleb formation on the endothelial surface, increased capillary permeability, interstitial edema, micro-thrombi, neutrophil adhesion and plugging, local acidosis, and presence of red blood cells with altered micro-rheology (impaired deformability and enhanced aggregation) [27,62,80,81,82,83,84,85]. Hemorheological and microcirculatory alterations in ischemia-reperfusion may show age- and gender-related differences as well [75,86,87].

In summary, the initiating effects (tissue damage, hypoxia, mechanical trauma to blood, free radicals, and their combinations) lead to metabolic alterations and inflammatory processes affecting micro- and macro-rheological parameters, flow characteristics and endothelial functions, resulting in microcirculatory disturbances and decrease in perfusion. These generate further tissue damage.

### Effect of Pringle/Baron Maneuver

Despite of the availability of modern devices controlling bleeding of parenchymal organs, temporarily clamping the hepatoduodenal ligament (Baron or Pringle maneuver) is still widely used in liver surgery for hepatic blood inflow control during major resections (open and laparoscopic/robotic surgery) to reduce bleeding and the need for perioperative blood transfusion [88,89,90,91,92]. Despite its obvious technical benefits during surgery, its use remains controversial due to potential negative effects on the clinical outcomes. Thanks to modern anesthesiology, intensive care, surgical devices and methods, the ischemic time tolerated by the liver has been extended in the past decades. Prolonged vascular inflow occlusion (≥60 min) can be safely applied using both continuous and intermittent regimens [93]. When performing the Pringle maneuver, besides hepatic ischemia, venous congestion also appears in the portal system, causing duration-dependent damage to the intestines as well [94].

Concerning hemorheological and microcirculatory changes, ischemia and reperfusion, congestion, hemoconcentration, metabolic alterations, all are important. In a canine model it has been demonstrated that the intermittent Pringle maneuver (three times, 15-min of ischemia alternating with 5-min of reperfusion) caused increased hematocrit and red blood cell aggregation in the systemic and hepatic venous blood samples immediately after the first maneuver. Followed by the second clamping, this increase was not seen, while after the third maneuver, red blood cell aggregation was enhanced again not only in the systemic, but also in the portal venous blood together with an increase in local hematocrit values [95].

## 5. Therapeutic Strategies

Prevention of IRI and its complications is still a largely unsolved issue in the clinical setting. To reduce IRI, numerous conditioning strategies including physical (hypothermia/cooling), pharmacological and surgical approaches (Table 1) have been tested in preclinical and clinical studies [8,10,96,97,98].

Pharmacological therapies were mostly explored in the setting of acute myocardial infarction, ischemic stroke and visceral IRI (liver, kidney, intestines). Pharmacological approaches include antioxidant agents, vasodilators, anti-inflammatory drugs, and agents that modify rheological parameters [17,104,105,106]. Although, many strategies such as remote ischemic conditioning (RIC) have shown promising results in the preclinical phase of testing, most of the clinical studies have failed to show a real clinical benefit [107].

Over the last 10 years, clinical machine perfusion of donor allografts before organ transplantation has been considered to be one of the most promising strategies to mitigate IRI and improve clinical outcomes in solid organ transplantation [4,9,99,100,108,109,110,111,112,113,114,115].

### 5.1. Ischemic Conditioning and Remote Conditioning

Described initially in the setting of myocardial infarction by Murry et al. [116], ischemic conditioning consists of brief periods of ischemia and reperfusion before target organ ischemia. Since its first introduction in 1986 a large body of evidence has accumulated on the effects of various pre-, post- and remote conditioning strategies (e.g., ischemic preconditioning (IPC); ischemic postconditioning (IPOST); remote conditioning (RIC)) [5,116]. Our groups and others have extensively investigated the effect of these IRI modulating therapies in various experimental models of liver IRI and OLT [5,99,115,117,118].

For 30 years, ischemic conditioning belonged to the major research topics of IRI research with dozens if not hundreds of research groups contributing to the understanding of the mechanisms and exploiting the potential benefits of this effective endogenous protective response. Mechanisms of IPC and RIC are still not completely understood and have been intensively discussed in previous comprehensive review articles [5,96,119,120].

In the field of clinical liver research, the best results have been reached by the application of IPC in the setting of major hepatectomies. In 2000, Clavien et al. provided the first clinical evidence suggesting the beneficial effects of IPC during major liver surgery [121]. In this pioneering study, 24 patients undergoing hemihepatectomy received an IPC protocol (10 min of ischemia and 10 min of reperfusion) before transection of the liver performed under inflow occlusion for exactly 30 min. IPC resulted in markedly reduced serum transaminase levels and in a dramatic reduction in the number of apoptotic sinusoidal lining cells. Interestingly, when IPC was applied in the setting of OLT as a donor therapy in a relatively large randomized control trial (RCT) with 101 deceased donors, it increased IRI without any adverse clinical consequences. However, IPC was associated with increased systemic levels of the anti-inflammatory cytokine IL-10 and fewer clinically important early rejections, defined as an “IPC paradox” [122]. Based on these encouraging results, some hepatic surgeons routinely use IPC or intermittent Pringle maneuver in complex partial hepatectomies, while many teams use hepatic inflow occlusion only “on demand” in selected cases [123,124,125].

Although some of these clinical findings with IPC are promising, the literature is partially contradicting. A systematic review and meta-analysis assessed the data of 669 patients from 11 RCTs using IPC in the setting of liver resection and failed to find a significant clinical benefit. A further meta-analysis on donor IPC in OLT demonstrated that IPC reduced liver injury following transplantation showing a tendency towards a reduced one-year mortality (6% vs. 11%, *p* = 0.06). During the past 10–15 years, most research groups were focusing on the effects of IPOST and RIC, which are clinically more feasible as they (in contrast to IPC) can also be used in acute clinical settings where the time point of the index ischemia is not known [119]. With rare exemptions, most pre-clinical studies demonstrated a well-reproducible and robust effect for all ischemic conditioning techniques including RIC in the setting of liver ischemia and liver transplantation [107]. Despite this large body of supporting preclinical evidence, clinical studies using especially the clinically most feasible RIC techniques often failed to show a real clinical benefit. The RIPCOLT trial by Robertson et al. has investigated the effects of RIC in a randomized trial with 40 OLT recipients [119]. Although, RIC has been confirmed to be safe, the authors could not demonstrate a reduction in liver injury. Similar findings have been obtained in other clinical scenarios including myocardial infarction [107] and kidney transplantation [101,126]. More promising but still heterogeneous and contradicting results were obtained in various IRI scenarios of the brain [127,128].

There are multiple factors behind the contradicting results and failure to clinically reproduce the robust protective effects of IPC, IPOST and RIC observed in preclinical studies [119,120]. While laboratory animals are in general healthy and have less genetic variability, in clinical settings, patients may have multiple co-morbidities, treated by a high-number of various drugs and presumably have a larger genetic variability of the IRI-related genes [118,119,120]. Obesity and diabetic neuropathy may interfere with the neural pathways involved in RIC induced protection [5,118,129]. Blood and plasma transfusions during surgery may result in a washout of important humoral factors and common anesthetic agents such as propofol also may interfere with the IPC and RIC triggered innate pathways [119,120,130].

Despite these difficulties and their limited clinical benefit, ischemic conditioning and remote conditioning still remain two of the most important techniques to explore the mechanisms of IRI and the protective mechanisms triggered by brief episodes of IR. This might help us to identify several molecular targets for pharmacological treatment.

Concerning the timing they can be used as pre-, per- and postconditioning, and protocols related to the target organ local (direct) or remote conditioning are known. The number and duration of the ischemic periods and the circumstances of reperfusion also differ depending on the target organ and even the examined species. Referring to the time interval between conditioning and target ischemia, delayed conditioning is also known. However, it might have different effects depending on the time interval (minutes, hours, or even a day before manifest ischemia). These processes activate three main types of protecting mechanisms against the ischemia-reperfusion injury: humoral (adenosine, l-arginine, eNOS, iNOS, bradykinin, opioids, HIF-1α, SDF-1α, GLP-1, apolipoprotein A1, microRNA144), systemic (macrophages, monocytes, T regs, pro-inflammatory cytokines, IL-10) and neural pathways (substance P, CGRP). The protection is manifested as reduced cell death and inflammatory response, and improved hepatic microcirculation [8,10,96,98,120,131,132].

Remote ischemic preconditioning (RIPC) is an alternative type of IPC when the preconditioning performed of another organ or extremity before the target organ ischemia-reperfusion. Both IPC and RIPC have an early and delayed window of protection. The early window lasts around 3 h after the IPC/RIPC, while the delayed window takes effect from 12 to 72 h after the conditioning [120]. Magyar et al., in a rat model, used a 60 min partial (~70%) liver ischemia followed by 120 min reperfusion and studied remote ischemic preconditioning (three cycles of 10 min ischemia and 10 min reperfusion) that was applied 1 h (“early-effect”) or 24 h (“delayed-effect”) before the liver ischemia. Microvascular perfusion of the liver increased in the “delayed-effect” group at the 120 min of the reperfusion. The red blood cell deformability improved better in the “early-effect” group, while erythrocyte aggregation index values decreased in both preconditioning groups [133]. However, a meta-analysis in 2019 found no benefits of RIPC in major vascular surgery [134].

In case of remote ischemic perconditioning the blood flow interruptions are performed at the same time as the manifest ischemia-reperfusion happens on the target organ, as firstly described by Schmidt et al. in 2007 [135]. A meta-analysis in 2017 found that perconditioning is a promising adjunctive treatment in case of patients with ST-elevation [136]. Experimental studies show usefulness in preventing hepatic IRI [102,137].

In clinical practice, IRI periods are often not planned. Zhao et al. in 2003 [138] described the ischemic postconditioning, when they performed three rounds of 30 s of reperfusion and 30 s ischemia just after 60-min of ischemia and found that this kind of local ischemic postconditioning reduced the size of the acute myocardial infarct in dogs [138]. However, a recent meta-analysis of patients who underwent primary percutaneous coronary intervention showed no effectiveness of ischemic postconditioning [139]. Li et al. suggested that a combined ischemic preconditioning and remote ischemic perconditioning strategy can be useful in the protection against ischemia-reperfusion injury in clinical liver transplantation [97].

Remote postconditioning was described by Kerendi et al. in 2005. They performed brief renal ischemia and reperfusion immediately before the onset of myocardial reperfusion and they found an almost 50% reduction in the myocardial infarct size [103]. It has been demonstrated that this method can be effective via the PI3K/ERK pathway [140].

Gradual reperfusion is a type of controlled reperfusion when the occluded or excluded artery is released carefully; therefore, the blood flow is gradually restored from zero to the uncontrolled level of autoregulation. The hypothesis is gradual reperfusion reduces the production of free radicals because it can confine the amount of oxygen and glucose. It is hard to precisely control the restoration of blood flow during each exclusion, even in the case of the same operator, the dynamics of release changes, so the extent of its effect can vary widely. This method of effectiveness is controversial and rarely examined [141,142,143]. It is important to note that nutritional status, diet, presence of steatosis or other hepatic alterations influence the effects of conditioning maneuvers and their outcome [144,145].

It should be noted that the increasing interest for dynamic organ preservation (machine perfusion) and pharmacological and genetic approaches has led to a reduced clinical focus on hepatic ischemic conditioning. This is well-depicted by the scarcity of interventional clinical trials with the use of ischemic conditioning in liver surgery and OLT. In our literature review we could only identify four completed clinical studies with rather controversial findings [146,147,148,149] (Table 2). Likewise, a recent search in two major trial databases found no registered clinical trials currently recruiting (accessed on 13 December 2020, https://www.clinicaltrials.gov and https://www.isrctn.com).

### 5.2. Pharmacological Strategies

There is a large body of pre-clinical data investigating the potential of dozens of various pharmacological agents in reducing hepatic IRI [9,89,150]. Although, many of these agents showed positive results in the preclinical testing and some have been used in clinical trials and even though some of these drugs showed positive effect on the extent of IRI in terms of the reduction hepatocellular injury, no single agent or “drug cocktail” has demonstrated a major clinical benefit which would justify its routine use [150].

Various groups have attempted to classify and categorize of agents of interest in reducing hepatic IRI as a first step in designing suitable multifactorial and pleiotropic approaches and develop pharmacological strategies with a high potential for clinical translation [150,151]. In a systematic review, Yamanaka et al. have identified and classified pharmacological agents according to their mechanisms of action: I—adenosine agonists, nitric oxide agonists, endothelin antagonists, and prostaglandins, II—Kupffer cell inactivators, III—complement inhibitors, IV—antioxidants, V—neutrophil inactivators, VI -anti-apoptosis agents, VII—heat shock protein and nuclear factor kappa B inducers, VIII—metabolic agents, IX—traditional Chinese medicine, and X—others [150]. Many of the previously tested agents belonging to the different categories target hemorheological and microcirculatory aspects of IRI. Group I agents are generally known to preserve and improve hepatic microcirculation. Agonists of the adenosine or nitric oxide pathways, endothelin antagonists and prostaglandins have the potential to mitigate the detrimental effects of post-ischemic microcirculatory failure [9,89,150]. In the early stages of reperfusion injury, microcirculatory damage is a pivotal mechanism in liver IR injury, microvascular injury plays an accentuated role in prolongation of the ischemic period and thus in aggravation of cellular injury, due to the “no-reflow” phenomenon on the level of the small hepatic sinusoids [62,152]. Main pathophysiological events of microcirculation dysfunction include the deterioration of active ion transport mechanisms, secondary to ischemia-induced ATP deficiency, and consequential cell swelling, cellular edema [152]. A consequential nitrogen oxide/endothelin imbalance and sinusoidal narrowing, during reperfusion are worsened by the accumulation of activated neutrophil cells and by reduced red blood cell velocity [151].

However, the above-mentioned Group I agents directly target various effectors of hepatic microcirculation reducing the detrimental effects of microvascular failure, multiple strategies and drugs exist to indirectly influence hepatic blood blow and microcirculation [9,151]. The correction of metabolic acidosis, modulation of inflammatory responses and immune cell activation and redox-household might have beneficial secondary effects on hepatic microcirculation as well [151,152].

### 5.3. Organ Preservation Techniques and Machine Perfusion

The standard practice of static cold storage (SCS) has changed very little over the past 40 years since the clinical introduction of University of Wisconsin (UW) solution [6,9]. Hypothermic preservation on ice is still a cornerstone of solid organ transplantation. Its availability, safety and feasibility has facilitated widespread application. Evidence shows that hypothermia reduces the tissue energy demand and extends the preservation period allowing even long-distance transport of donor allografts. Since the introduction of UW, a number of newer organ preservation solutions have been developed such as Histidine–tryptophan–ketoglutarate (HTK) solution, Institute Georges Lopez-1 (IGL), Leeds solution (LS), and POLYSOL as well as ECOSOL which have been intensively tested by our author team and by others [153,154,155,156,157,158]. However, all the above-mentioned preservation solutions represent a certain improvement in their composition compared to the original UW solution with potential benefits in various transplant scenarios, none of these solutions have led to a major improvement in clinical outcomes of liver transplantation [157].

In the face of the critical organ shortage of the recent years, dynamic ex situ organ preservation and reconditioning techniques gained an increasing interest in solid organ transplantation [4,6,115]. It has been increasingly recognized that marginal allografts poorly tolerate extended periods of static cold storage. Currently, two main paradigms prevail in clinical liver machine perfusion. Hypothermic machine perfusion (HMP) is mainly seen as a modern dynamic variant of traditional hypothermia-based organ preservation and, due to its technical feasibility and simplicity, both HMP and hypothermic oxygenated machine perfusion (HOPE) are increasingly used by different groups around the globe. Meanwhile, normothermic or subnormothermic machine perfusion (NMP) aim to avoid or minimize cold ischemic injury by providing nearly physiological conditions, oxygen and nutrients. The first large multicenter machine perfusion RCT in liver transplantation compared the effects of NMP with SCS and completed recruitment in 2016. The primary endpoint of the study published in Nature, peak AST, was significantly reduced by NMP compared to SCS accompanied by a lower discard rate in the NMP group [159]. Recently, the Zurich group has arrived at a refined advanced NMP setting that allowed a 7-day preservation of human livers with sustained metabolic function and intact liver structure [160]. Table 3 overviews ongoing randomized clinical trials.

Although, there has been some research on the rheological and circulatory aspects of various dynamic organ preservation approaches, the exact role of these mechanisms in the HMP and NMP induced protection remains to be elucidated. Burlage et al. have used 18 declined livers for transplantation and demonstrated an improved endothelial cell function in marginal allografts via the upregulation of mechanosensitive (shear stress) cytoprotective genes which resulted in better preservation of endothelial cell morphology [161]. Although multiple authors confirmed endothelial shear stress as one of the main mechanism of machine perfusion that induced protection compared to static cold storage, some studies have also emphasized the detrimental effects and risks of higher perfusion flow and pressure, leading to sinusoidal endothelial cell injury with subsequent activation of Kupffer- and endothelial cells [162]. Therefore, current clinical devices mostly use pressure-controlled perfusion to minimize the risk of increased shear stress related graft damage [161,162].

Supporting data have also accumulated in liver machine perfusion using NMP. Goldarecena et al. have observed improved endothelial function in porcine liver transplantation model with the utilization of NMP in a combination with anti-inflammatory perfusate additives [163,164].

## 6. Future Perspectives and Remaining Challenges

Over the last 20 years, significant advances have been made in the field of liver surgery and transplantation. IRI remains one of the leading issues in solid organ transplantation and in major hepatic resection with inflow occlusion. Despite the large and continuously increasing interest for IRI in basic research, there is still only limited success regarding clinical translation of various experimental therapies (Figure 1).

In the future, the better and more complex understanding of the mechanisms of IRI could help us not only to better design clinical trials but also in the identification of novel therapeutic targets and techniques [4,6,162,165,166,167,168,169,170,171,172,173,174,175,176,177,178,179,180,181,182] (Table 4). Gene therapy (e.g., genetic preconditioning) is one of the novel approaches finding its way into IRI therapy [151]. In hepatic IRI mainly viral vectors have been used to modulate various genes leading to scavenging ROS or increasing detoxifying capacity [151]. High doses of mitochondrial superoxide dismutase (SOD) enzyme administered via viral vector in mice has led to a significant reduction in IRI [151,183].

Machine perfusion in solid organ transplantation is increasingly in the spotlight of scientific interest [4,6,184]. In the recent years, multiple studies have confirmed the beneficial clinical effects of machine perfusion in the setting of liver transplantation [4,6]. Currently various larger RCTs with the use of HMP, HOPE and NMP are recruiting or have finished recruitment. The results of these important clinical studies will help our understanding of how MP influences clinical outcome in OLT and hopefully will lead to a major paradigm shift in the clinical practice of the coming years [4,6,184]. Machine perfusion will also provide a unique platform to deliver various ex vivo therapies without major systemic effects. Several laboratories have already investigated the benefits and limitations of allograft defatting, gene modulating agents, cytokine filtration, anti-inflammatory drugs, stem cells and extracellular vesicles as well as vasodilators in the setting of ex vivo machine perfusion [185]. Furthermore, machine perfusion driven viability assessment during HMP/HOPE or NMP may support clinical decision in the acceptance or rejection of marginal liver allografts [4,6,184]. Besides refinement of the preservation techniques, the impact of hepatic dysfunction on macro- and micro-rheological parameters still need to be investigated, including metabolomics studies, to reveal better the local and systemic effects [186,187,188,189,190,191,192,193]. However, it would be interesting to overview it in a separate paper.

## 7. Conclusions

In conclusion, hemorheological factors play an important role determining tissue perfusion and orchestrate mechanical stress-dependent endothelial functions. During IRI dominantly non-specific alterations lead to deteriorating micro-rheological properties as well. Antioxidant and anti-inflammatory agents, ischemic conditioning protocols and machine perfusion may improve rheological properties in the hepatic sinusoids contributing to the protection against hepatic IRI. Studying hemorheological factors together with microcirculatory investigations may provide useful information for better understating the pathomechanism of hepatic ischemia-reperfusion and to optimize surgical conditioning protocols and organ preservation techniques.

## Figures and Tables

**Table 1:** Ischemic conditioning strategies related to index hepatic ischemia-reperfusion injury (IRI).

Ischemic Conditioning Strategy ^§^	
Preconditioning	Local/target organ (early, delayed) *
	Remote organ ^#^ (early, delayed) *
Perconditioning	Local/target organ
	Remote organ
Postconditioning	Local/target organ
	Remote organ
Gradual perfusion	Local/target organ
Combined methods	e.g., Local ischemic pre- and postconditioning, local ischemic preconditioning and remote organ perconditioning

* early and delayed effects based on the timing before ischemia manifestation; ^#^ remote organ: limb, intestine, kidney [99,100,101,102,103]; ^§^ there is a major heterogeneity in the literature concerning the abbreviations and nomenclature of various ischemic conditioning approaches.

**Table 2:** Clinical studies using remote ischemic condition in liver transplantation and partial hepatectomies.

Author	Study Type	Patients	Type of RIC	Follow-Up	Outcome and Conclusion
Robertson et al. [146]	Randomized controlled pilot study	40 patients receiving LT	in the recipient, 3 × 5 min IR, before surgery, left leg pneumatic tourniquet	90 days follow-up	No significant difference in median AST levels. No reduction in short-term outcomes.
Wu et al. [147]	Randomized controlled trial	120 patients undergoing partial hepatectomy (control vs. IPC vs. RIPC)	3 × 5 min IR, before surgery, right arm	n.r.	Significant decrease in serum ALT and AST levels in IPC and RIPC groups. Significant decrease in TWEAK in IPC group.
Kim et al. [148]	Randomized controlled trial	78 patients undergoing living donor LT	4 × 5 min, IR after reperfusion of the transplanted liver, one arm	6 months follow-up	No difference in graft function or clinical outcomes. Decreased incidence of AKI in RIC.
Teo et al. [149]	Randomized controlled trial	50 patients undergoing partial hepatectomy	4 × 5 min, before surgery, one arm	n.r.	No reduction in serum ALT levels. No clinical benefits were observed after RIC.

Accessed: 13 December 2020. Abbreviations used: IR—ischemia reperfusion; IPC—ischemic preconditioning; RIC—remote ischemic conditioning; RIPC—remote ischemic preconditioning; RIPostC—remote ischemic postconditioning; LT—liver transplantation; TWEAK—tumor necrosis factor-like weak inducer of apoptosis; HIRI—hepatic ischemic reperfusion injury; AST—aspartate aminotransferase; ALT—alanine aminotransferase; AKI—acute kidney injury; n.r.—not reported.

**Table 3:** Overview of currently ongoing prospective randomized clinical trials on ex vivo machine perfusion in liver transplantation.

Study/Author	Design	MP Modality	Allografts	Target Sample Size	Recruitment Status
TransMedics, Inc., Andover, MA, USA NCT02522871	Multicenter RCT	NMP using the OCS™ Liver system vs. SCS	n.r.	300	Active, not recruiting
Durham, NC, USA NCT02775162	Multicenter RCT	NMP vs. SCS	n.r.	267	Active, not recruiting
Lyon, France NCT03929523	Multicenter, RCT	HOPE vs. SCS	ECD-DBD	266	Recruiting
Berlin, Germany NCT04644744	Multicenter RCT	NMP vs. HOPE vs. SCS	ECD-DBD	213	Recruiting
Zurich, Switzerland NCT01317342	Multicenter, RCT	HOPE vs. SCS	DBD	170	Completed
Groningen, Netherlands NCT02584283	Multicenter RCT	Dual HOPE vs. SCS	DCD	157	Completed
Newark, NJ, USA NCT03484455	Multicenter RCT	HMP vs. SCS	n.r.	140	Recruiting
Bologna, Italy NCT03837197	Multicenter RCT	HOPE vs. SCS	ECD-DBD	110	Recruiting
Aachen, Germany NCT03124641	Multicenter, RCT	HOPE vs. SCS	ECD-DBD	46	Completed
Essen, Germany ISRCTN94691167	Single-center pilot RCT	COR vs. SCS	SC-DBD	40	Recruiting

Screening: 10 February 2021. MP—machine perfusion; ECD—extended criteria donor; DBD—donation after brain death; DCD—donation after circulatory death; SCS—static cold storage; NMP—normothermic machine perfusion; HMP—hypothermic machine perfusion; HOPE—hypothermic oxygenated machine perfusion; COR—controlled oxygenated rewarming; SC—standard criteria; RCT—randomized controlled trial; n.r.—not reported.

**Table 4:** Future directions in liver ischemia-reperfusion and organ preservation research.

Research Direction	Details and Subtopics	Reference
Innovations in dynamic and static liver preservation	Acellular oxygen carriers for NMP. Combined preservation techniques (e.g., SCS–HOPE–COR–NMP). Novel preservation/perfusion solutions. Reconditioning with gaseous substances. Combination NRP with NMP or HMP/HOPE. Ischemia-free liver transplantation. Comparison of effects, safety and feasibility of MP modalities (e.g., HOPE vs. NMP, HOPE vs. HMP). Ex situ on pump liver splitting during HMP/HOPE/NMP. Supercooling. Super-extended machine preservation (e.g., 7-days and longer).	[4,6,162,165,166,167,168,169]
Therapeutics and pharmacological conditioning	Revitalization of steatotic livers through defatting agents. Conditioning through of gas. Stem cell-derived therapy. Extracellular vesicles. Gene therapy (e.g., gene silencing with RNAi). Antiviral treatment during MP. Pharmacological agents targeting IRI and microcirculation regulatory pathways, administered during machine perfusion or as donor/recipient therapy.	[4,6,89,170,171,172,173,174,175,176,177,178,179]
Allograft viability assessment	Perfusate, bile, tissue biomarkers of viability. New analytical technologies e.g., proteomics and metabolomics.	[180,181,182]

Abbreviations used: IRI—ischemic reperfusion injury; MP—machine perfusion; HMP—hypothermic machine perfusion; HOPE—hypothermic oxygenated machine perfusion; NMP—normothermic machine perfusion; NRP—normothermic regional perfusion; SCS—static cold storage.

## Data Availability

Data sharing not applicable.

## Abbreviations

DBD	Donation after brain death
EAD	Early allograft dysfunction
ECM	Extracellular matrix
HMP	Hypothermic machine perfusion
HOPE	Hypothermic oxygenated machine perfusion
HSC	Hepatic stellate cells
HTK	Histidine-tryptophan-ketoglutarate solution
IPC	Ischemic preconditioning
IPOST	Ischemic postconditioning
IRI	Ischemia-reperfusion injury
KC	Kupffer cells
MP	Machine perfusion
MPS	Mononuclear phagocyte system
NMP	Normothermic or subnormothermic machine perfusion
NO	Nitric oxide
OLT	Orthotopic liver transplantation
RBC	Red blood cell
RCT	Randomized clinical trial
RIC	Remote conditioning
ROS	Reactive oxygen species
SCS	Static cold storage
SEC	Sinusoidal endothelial cells
SOD	Superoxide dismutase
UW	University of Wisconsin solution
